# Preoperative computed tomography for assessment of bone invasion in oral squamous cell carcinoma: diagnostic accuracy and anatomical subsite dependency

**DOI:** 10.1007/s00432-026-06472-2

**Published:** 2026-04-22

**Authors:** Jannik Ketschau, Yannik Leonhardt, Alex Grabenhorst, Hannes Singer, Helena Kram, Nils Krautkremer, Cornelius Leopold, Jonathan Mohr, Herbert Stimmer, Klaus-Dietrich Wolff, Lucas M. Ritschl

**Affiliations:** 1 Department of Oral and Maxillofacial Surgery, TUM University Hospital Klinikum Rechts der Isar, School of Medicine and Health, Technical University of Munich, Munich, Germany; 2 Institut of Diagnostic und Interventional Radiology, TUM University Hospital Klinikum Rechts der Isar, School of Medicine and Health, Technical University of Munich, Munich, Germany

**Keywords:** Oral squamous cell carcinoma, Bone invasion, Computed tomography, Diagnostic accuracy, Staging, Radiological assessment

## Abstract

**Purpose:**

Accurate preoperative assessment of bone invasion is crucial in oral squamous cell carcinoma (OSCC), because it directly influences staging and surgical planning. Computed tomography (CT) is widely used, but its diagnostic performance may vary with tumor localization and image quality.

**Methods:**

In this retrospective study, patients with OSCC who underwent preoperative CT imaging and subsequent surgical resection with histopathological evaluation were identified. Bone invasion was assessed on CT using a graded and dichotomous classification. Histopathology served as reference standard. Diagnostic performance was analyzed overall and stratified by tumor localization, radiological severity, and image quality.

**Results:**

572 patients were included. Histologically confirmed bone invasion was present in 134 cases (23.6%). Overall, CT demonstrated a sensitivity of 63.4% and a specificity of 90.8%, with an overall diagnostic accuracy of 84.3%. The probability of bone invasion increased stepwise with increasing radiological severity (*p* < 0.001). Diagnostic performance varied by tumor localization, with significant differences in specificity and overall accuracy (*p* < 0.001), while sensitivity did not differ significantly (*p* = 0.597). Radiological grading correlated with pathological stage; nevertheless, 12.3% of pT2 tumors were interpreted as showing bone involvement, while 36.6% of histologically confirmed pT4 tumors remained radiologically occult. Exclusion of cases with relevant imaging artifacts resulted in improved diagnostic performance (AUC 0.80 vs. 0.78).

**Conclusion:**

Preoperative CT provides clinically relevant information for assessing bone invasion in OSCC. However, diagnostic performance varies across anatomical subsites, as well as radiological severity and image quality. A localization-aware interpretation may help to avoid under- and overtreatment.

**Supplementary Information:**

The online version contains supplementary material available at 10.1007/s00432-026-06472-2.

## Introduction

Accurate preoperative assessment of bone invasion is a critical component in the management of oral squamous cell carcinoma (OSCC). The presence of bone infiltration has major implications for tumor staging, surgical planning, extent of resection, and functional outcome (Ebrahimi et al. 2011; Fives et al. 2017; Li et al. 2017; Ritschl et al. 2023). Inadequate assessment may either lead to insufficient oncologic clearance or to overtreatment with unnecessary bone resection, both of which are associated with significant morbidity and impairment of life quality (Okura et al. 2016; DeAngelis et al. 2019; Lee et al. 2020).

Computed tomography (CT) is widely regarded as the primary imaging modality for evaluating suspected bone involvement in OSCC, due to its broad availability and high spatial resolution for cortical bone assessment (Handschel et al. 2012; Slieker et al. 2020; Struckmeier et al. 2024). Radiological signs such as cortical erosion or bone destruction are commonly used to guide surgical decision-making. However, the diagnostic accuracy of CT for detecting bone invasion remains imperfect, particularly in early or microscopic disease (Li et al. 2014; Kouketsu et al. 2021; Gill et al. 2025).

Previous studies have reported variable sensitivity and specificity of CT for bone invasion, reflecting heterogeneity in patient populations, tumor subsites, radiological criteria, and reference standards (Hakim et al. 2014; Li et al. 2014). Importantly, the anatomical relationship between the primary tumor and adjacent bone differs substantially across oral subsites, suggesting that the diagnostic performance of CT may not be uniform across all tumor locations. Nevertheless, localization-specific diagnostic accuracy has not been systematically addressed in large, histologically validated cohorts (Slieker et al. 2020; Struckmeier et al. 2024).

The aim of the present study was therefore to evaluate the diagnostic performance of preoperative CT for the detection of histologically confirmed bone invasion in a large cohort of patients with OSCC. Specifically, we sought to (i) assess overall diagnostic accuracy, (ii) examine the relationship between radiological severity and pathological bone invasion, (iii) analyze the impact of imaging artifacts, and (iv) investigate how diagnostic performance varies according to tumor localization.

## Materials and methods

### Ethical considerations, study design and patient cohort

The study was conducted in accordance with the Declaration of Helsinki. Approval was obtained from the institutional ethics committee prior to data collection (approval number: 2022-234-S-KH). Due to the retrospective nature of the study and the use of anonymized data, the requirement for informed consent was waived.

This retrospective single-center study included patients treated for primary OSCC between 2011 and 2021 in the department of oral and maxillofacial surgery, University Hospital, Technical University of Munich, Germany. Inclusion criteria were availability of a preoperative, in-house performed contrast-enhanced CT scan of the head and neck, definitive surgical resection of the primary tumor and histopathological assessment of bone invasion. Patients with recurrent or secondary malignancies or without evaluable CT imaging, external performed CT-imaging or with missing histological data regarding bone infiltration were excluded.

### Radiological assessment

**Fig. 1:**
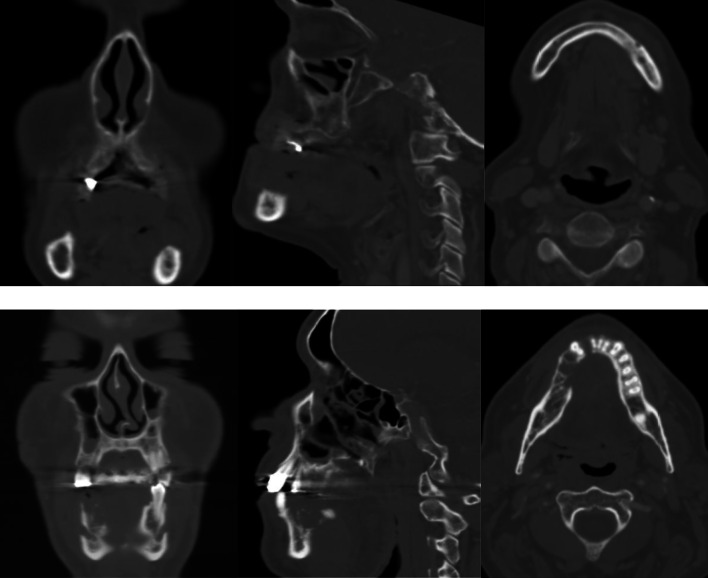
CT-based grading of bone involvement. Representative CT images illustrating cortical erosion (upper row, left mandible) and bone destruction (lower row, right mandible) of the mandible in frontal (left), sagital (middle) and axial (right) plane

All preoperative CT examinations were reviewed based on the original radiology reports, extracted out of the hospitals PACS (picture archiving and communication system), carried out by at least two board certified radiologist with substantial experience in head and neck imaging (Fig. 1). Radiological assessment of bone involvement was categorized using an ordinal grading system:

- No bone invasion/not mentioned
- Cortical erosion
- Bone destruction

Radiological findings were additionally dichotomized as negative (no bone affection/not mentioned) or positive (cortical erosion or bone destruction). The graded classification was used for severity analyses and ROC analysis, whereas the dichotomous classification was used to calculate sensitivity, specificity, PPV, NPV, and accuracy.

The presence of relevant imaging artifacts potentially interfering with bone assessment (e.g., dental artifacts) was recorded as a binary variable (present vs. absent). Sensitivity analyses were performed after exclusion of artifact-affected scans.

### Histopathological assessment

Clinical and pathological data were extracted from the electronic patient records of the hospital information system (SAP). Histopathological evaluation of surgical specimens served as the reference standard. Bone invasion was defined as histologically confirmed tumor infiltration into cortical and/or medullary bone. Histopathological assessment was performed according to institutional standards by experienced head and neck pathologists. In patients without bone resection, bone invasion was considered absent if there was no histological or intraoperative evidence of bone involvement.

### Tumor localization

Primary tumor localization was classified into the following anatomical subsites: floor of the mouth, tongue, mandibular alveolar ridge, maxillary alveolar ridge, buccal mucosa, retromolar region, hard palate, and soft palate. Localization-specific analyses were performed to assess differences in diagnostic performance across subsites.

### Statistical analysis

Descriptive statistics were used to summarize patient characteristics, tumor localization, radiological findings, and histopathological outcomes. Categorical variables are presented as frequencies and percentages, while continuous variables are reported as mean ± standard deviation or median with minimum–maximum range, as appropriate.

Diagnostic accuracy of CT for detection of bone invasion was evaluated using sensitivity, specificity, positive predictive value (PPV), negative predictive value (NPV), and overall accuracy, with histopathology as the reference standard. Associations between radiological findings and bone invasion were assessed using chi-square or Fisher’s exact tests, as appropriate.

Receiver operating characteristic (ROC) analysis was performed to evaluate the discriminative ability of graded radiological findings, with calculation of the area under the curve (AUC). The optimal diagnostic threshold was determined using the Youden index.

Sensitivity analyses were conducted after exclusion of cases with relevant imaging artifacts. To compare localization-specific diagnostic performance, sensitivity was analyzed among patients with histologically confirmed bone invasion and specificity among patients without histological bone invasion. Differences across subsites were assessed using chi-square tests. In addition, diagnostic accuracy was evaluated using a dichotomous variable indicating correct versus incorrect classification, and compared across subsites by chi-square testing. Due to small sample sizes in selected subsites, these analyses were interpreted with caution and considered exploratory.

All statistical analyses were performed using IBM SPSS Statistics (version 29). A two-sided *p*-value < 0.05 was considered statistically significant.

## Results

### Study population

**Fig. 2:**
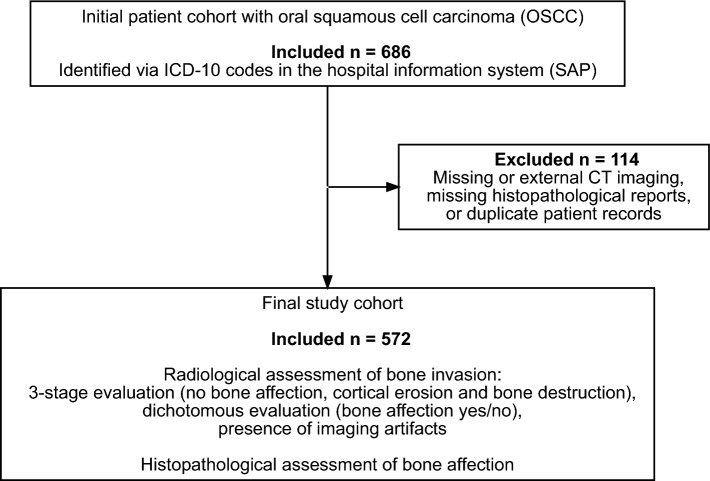
Flowchart of patient selection. Flowchart illustrating patient selection and data extraction for the study cohort

A total of 572 patients with primary OSCC were included in the analysis (Fig. 2). The median age was 63.0 (18.7–91.0) years, and 62.5% of patients were male. Most patients were classified as ASA II (71.3%) or ASA III (21.9%). Detailed patient characteristics are summarized in Table 1.

**Table 1:** Baseline patient characteristics

Characteristic	Total cohort (n = 572)
*Patient characteristics*	
Age (years)	63.0 (18.7–91.0)
Sex (male)	357 (62.5%)
ASA score	
ASA I	39 (6.8%)
ASA II	408 (71.3%)
ASA III	125 (21.9%)
Smoking status (current smoker)	313 (54.7%)
Diabetes mellitus	45 (7.9%)
Alcohol consumption	164 (28.8%)
Body mass index (kg/m^2^)	25.2 ± 4.4
*Tumor characteristics*	
Tumor localization	
Floor of the mouth	212 (37.1%)
Tongue	155 (27.1%)
Mandibular alveolar ridge	98 (17.1%)
Buccal mucosa	46 (8.0%)
Retromolar region	18 (3.1%)
Maxillary alveolar ridge	22 (3.8%)
Hard palate	6 (1.0%)
Soft palate	15 (2.6%)
Depth of invasion (DOI) (mm)	6.0 (0–50)
*Pathological T stage*	
pT1	211 (36.9%)
pT2	163 (28.5%)
pT3	62 (10.8%)
pT4	136 (23.8%)
*Radiological findings (CT)*	
Radiological grading	
No bone affection / not mentioned	447 (78.1%)
Cortical erosion	51 (8.9%)
Bone destruction	74 (12.9%)
*Radiological assessment (dichotomous)*	
Negative	447 (78.1%)
Positive (erosion or destruction)	125 (21.9%)
Imaging artifacts present	117 (20.5%)
*Histopathological findings*	
Histologically confirmed bone invasion	134 (23.6%)

Baseline characteristics of the study cohort, including patient demographics, tumor localization, radiological CT findings, and histopathological assessment of bone invasion

Tumors most frequently originated from the floor of the mouth (37.1%) and tongue (27.1%), followed by the mandibular alveolar ridge (17.1%). Histologically confirmed bone invasion was present in 134 of 572 evaluable patients (23.6%).

On preoperative CT imaging, radiological assessment indicated no evidence of bone involvement or no mention of bone affection in 447 patients (78.1%), while cortical erosion and bone destruction were reported in 51 (8.9%) and 74 patients (12.9%), respectively; when dichotomized, radiological bone involvement was present in 125 cases (21.9%).

### Distribution of bone invasion by tumor localization

The prevalence of histologically confirmed bone invasion varied markedly across anatomical subsites (supplementary Table 1). Bone invasion was most frequently observed in tumors of the maxillary alveolar ridge (77.3%) and mandibular alveolar ridge (51.0%). Intermediate rates were seen in tumors of the floor of the mouth (23.1%) and retromolar region (27.8%), whereas bone invasion was rare in tongue carcinomas (2.6%).

### Radiological assessment of bone invasion

On preoperative CT imaging, radiological signs of bone involvement were reported in 125 of 572 patients (21.9%). Using a graded radiological classification, no evidence of bone invasion was reported in 447 cases (78.1%), cortical erosion in 51 cases (8.9%), and bone destruction in 74 cases (12.9%).

The distribution of radiological findings differed by tumor localization (supplementary Table 2). Radiological bone involvement was most frequently reported in tumors of the alveolar ridge, particularly the maxillary alveolar ridge (68.2%) and mandibular alveolar ridge (50.0%). In contrast, radiological bone involvement was rarely detected in tongue carcinomas (1.3%).

### Diagnostic accuracy of CT for detection of bone invasion

Using histopathology as the reference standard, preoperative CT demonstrated a sensitivity of 63.4% and a specificity of 90.8% for the detection of bone invasion. The positive and negative predictive values were 68.0% and 88.9%, respectively, resulting in an overall diagnostic accuracy of 84.3% (Table 2). A strong association between radiological suspicion of bone invasion and histological confirmation was observed (χ^2^ = 175.3, *p* < 0.001).

**Table 2:** Diagnostic performance of computed tomography

Diagnostic parameter	Value
Sensitivity	63.4%
Specificity	90.8%
Positive predictive value	68.0%
Negative predictive value	88.9%
Overall accuracy	84.3%
Odds ratio (CT positive)	17.1 (95% CI 10.6–27.6)
Chi-square test	χ^2^ = 175.3
*p*-value	< 0.001

Diagnostic performance parameters of preoperative computed tomography for detection of histologically confirmed bone invasion in oral squamous cell carcinoma. Sensitivity, specificity, positive predictive value, negative predictive value, overall accuracy, odds ratio, and chi-square statistics are reported. Histopathology served as the reference standard

### Radiological severity and probability of bone invasion

Stratification by radiological severity revealed a pronounced stepwise increase in the probability of histologically confirmed bone invasion (supplementary Table S3). Bone invasion was present in 11.1% of cases without radiological signs of invasion, increased to 49.0% in cases with cortical erosion, and reached 81.1% in cases with overt bone destruction (*p* < 0.001).

**Table 3:** Association of CT-assessed bone involvement and T-stage

Histologic bone invasion	pT stage	No bone affection	Cortical erosion	Bone destruction	Total	*p*-value
Absent (n = 438)	pT1 (n = 211)	204 (96.7%)	4 (1.9%)	3 (1.4%)	211	
	pT2 (n = 163)	143 (87.7%)	15 (9.2%)	5 (3.1%)	163	
	pT3 (n = 62)	51 (82.3%)	6 (9.7%)	5 (8.1%)	62	
	pT4 (n = 2)	0 (0%)	1 (50.0%)	1 (50.0%)	2	
Present (n = 134)	pT4 (n = 134)	49 (36.6%)	25 (18.7%)	60 (44.8%)	134	
						** < 0.001**

Distribution of radiological bone involvement (no bone affection, cortical erosion, bone destruction) according to pathological T stage and histologically confirmed bone invasion in oral squamous cell carcinoma. Percentages refer to row proportions within each T stage. Histopathology was used as the reference standard

**Fig. 3:**
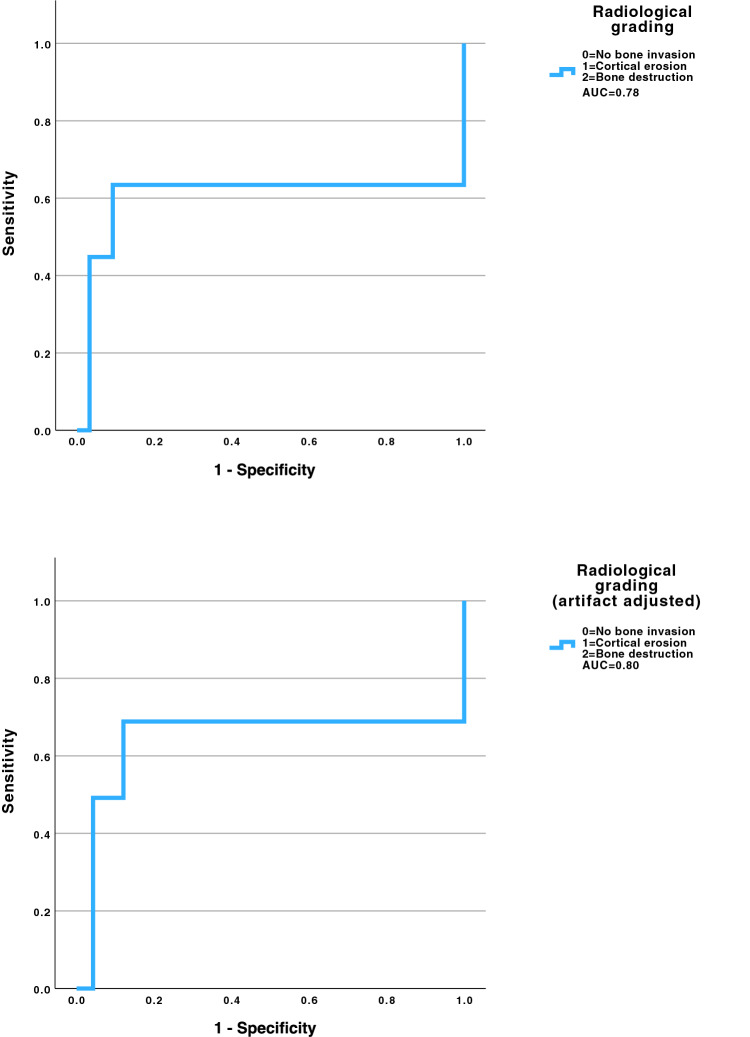
Diagnostic performance adjusted to artefacts. Receiver operating characteristic (ROC) curve illustrating the diagnostic performance of radiological findings (upper graph) and artifact-adjusted radiological grading (lower graph) for detection of histologically confirmed bone invasion in oral squamous cell carcinoma. Radiological severity was classified as no bone invasion, cortical erosion, or bone destruction. The area under the curve (AUC) was 0.78 and improved to 0.80 for artifact adjusted grading

Receiver operating characteristic analysis of the graded radiological assessment yielded an area under the curve (AUC) of 0.78, indicating good discriminative ability (Fig. 3).

Among tumors without histological bone invasion (n = 438), the majority were classified as pT1 (n = 211), pT2 (n = 163), or pT3 (n = 62). In pT1 tumors, 204 cases (96.7%) showed no radiological signs of bone involvement, while cortical erosion and bone destruction were reported in 4 (1.9%) and 3 cases (1.4%), respectively. In pT2 tumors, 143 cases (87.7%) were radiologically negative, whereas 15 (9.2%) demonstrated cortical erosion and 5 (3.1%) bone destruction. Among pT3 tumors, 51 cases (82.3%) showed no radiological bone affection, with 6 (9.7%) exhibiting cortical erosion and 5 (8.1%) bone destruction. Only two tumors without histological bone invasion were classified as pT4; in these cases, one demonstrated cortical erosion and one bone destruction on CT imaging. All tumors with histologically confirmed bone invasion (n = 134) were classified as pT4. Within this subgroup, 60 tumors (44.8%) demonstrated radiological bone destruction, 25 tumors (18.7%) showed cortical erosion, and 49 tumors (36.6%) exhibited no radiological signs of bone involvement (Table 3).

### Sensitivity analysis excluding imaging artifacts

Relevant imaging artifacts were present in 117 patients (20.5%). After exclusion of these cases, 455 patients remained for the sensitivity analysis. Sensitivity increased to 68.9%, while specificity remained high at 87.9%. The positive and negative predictive values were 67.7% and 88.4%, respectively. ROC analysis in this subgroup yielded an AUC of 0.80 (supplementary Table S4, Fig. S1).

### Localization-specific diagnostic performance

Diagnostic performance of CT varied across anatomical subsites (Table 4). Among patients with histologically confirmed bone invasion (n = 134), sensitivity did not differ significantly between subsites (*p* = 0.597). In contrast, among patients without histological bone invasion (n = 434), specificity differed significantly across subsites (*p* < 0.001). Specificity was highest in tongue carcinomas (99.3%) and lowest in tumors of the maxillary alveolar ridge (40.0%).

**Table 4:** Diagnostic performance dependent on tumor localization

Tumor localization	Sensitivity	Specificity	PPV	NPV	Accuracy
Floor of the mouth	63.3%	92.0%	70.5%	89.3%	85.4%
Tongue	25.0%	99.3%	50.0%	98.0%	97.4%
Mandibular alveolar ridge	66.0%	66.7%	67.3%	65.3%	66.3%
Buccal mucosa	57.1%	89.5%	50.0%	91.9%	84.4%
Retromolar region	60.0%	100%	100%	86.7%	88.9%
Maxillary alveolar ridge	70.6%	40.0%	80.0%	28.6%	63.6%
*p*-value	0.597	** < 0.001**	–	–	** < 0.001**

Sensitivity, specificity, positive predictive value (PPV), and negative predictive value (NPV) of preoperative computed tomography (CT) for detection of histologically confirmed bone invasion, calculated separately for each tumor localization. Histopathology served as the reference standard.

Bold *p*-values indicate statistical significance (p < 0.05).

Accordingly, overall diagnostic accuracy also varied significantly by tumor localization (*p* < 0.001). Accuracy was highest in tongue carcinomas (97.4%) and lower in tumors of the mandibular and maxillary alveolar ridge (66.3% and 63.6%, respectively). Due to small sample sizes, results for tumors of the hard palate, soft palate, and retromolar region should be interpreted with caution.

## Discussion

The aim of this study was to evaluate the diagnostic performance of preoperative CT for the detection of histologically confirmed bone invasion in OSCC, with particular emphasis on radiological severity, tumor localization, and image quality.

In this large, histologically validated cohort of 572 patients, CT demonstrated a sensitivity of 63.4%, a specificity of 90.8% and an overall diagnostic accuracy of 84.3% for the detection of bone invasion. The probability of histologically confirmed bone invasion increased stepwise with radiological severity, from 11.1% in cases without radiological signs of bone involvement to 49.0% in cases with suspicion of at least cortical erosion and 81.1% in cases with overt bone destruction (*p* < 0.001). Diagnostic performance varied substantially by tumor localization. While sensitivity did not differ significantly across subsites (*p* = 0.597), specificity and overall diagnostic accuracy showed significant variation (both *p* < 0.001), indicating that subsite-dependent differences are primarily driven by variation in specificity. Highest specificity was observed in tongue carcinomas (99.3%), whereas lower specificity was found in tumors of the maxillary alveolar ridge (40.0%) and mandibular alveolar ridge (66.7%). Exclusion of cases with relevant imaging artifacts resulted in a modest improvement in diagnostic performance, increasing sensitivity to 68.9% and the area under the ROC curve from 0.78 to 0.80.

In contrast to studies based on retrospective image re-evaluation or focused study protocols (Tudor and Finlay 1999), our analysis reflects routine clinical reporting and therefore real-world practice. While this enhances the generalizability of our results, it may partly explain the moderate sensitivity observed, particularly for subtle or early bone invasion that is more likely to be detected under study-driven conditions (Onoue et al. 2021; Haseli et al. 2025; Timmer et al. 2025).

In our cohort, the probability of histologically confirmed bone invasion increased stepwise with radiological severity, ranging from a low prevalence in tumors without radiological signs of bone involvement to nearly half of cases with cortical erosion and more than 80% of cases with overt bone destruction. This pronounced gradient suggests that cortical erosion represents an intermediate radiological stage and is consistent with previous studies reporting a stepwise correlation between radiological severity on CT and histologically confirmed bone invasion in OSCC, with increasing radiological severity reflecting deeper and more extensive pathological involvement (Goerres et al. 2005; Ebrahimi et al. 2011; Uribe et al. 2013). According to the 8th edition of the TNM classification, cortical bone erosions are currently classified as pT2-stage tumors (Amin et al. 2017). This categorization, together with reliable pretherapeutic assessment, has significant implications for subsequent treatment strategies. In these cases, a box resection is considered oncologically sufficient, and no adjuvant radiotherapy is required postoperatively (Ritschl et al. 2023). Although cortical erosion or even bone destruction was occasionally described radiologically in pT2 tumors, these findings were not confirmed histopathologically. This suggests that radiological bone irregularities in intermediate-stage tumors may reflect reactive or anatomical changes rather than true tumor infiltration. In contrast, pT4 tumors demonstrated a markedly different pattern, with a high prevalence of radiological bone destruction and universal histological bone invasion. However, more than one third of pT4 tumors were radiologically occult, indicating that absence of CT-based bone involvement cannot reliably exclude advanced disease.

These considerations are particularly important in the pretherapeutic setting, as they form a crucial part of the informed consent discussion and enable optimal patient counseling regarding the anticipated clinical course. Moreover, this information also has relevant health-economic implications. Patients undergoing box resection generally demonstrate shorter hospital stays and reduced intensive care unit duration compared with patients requiring continuity resection, which necessitates reconstruction using various techniques (Ritschl et al. 2023).

### Influencing factors

The most important contribution of this study is the demonstration that CT performance varies substantially by tumor localization (Arya et al. 2014; Gill et al. 2025). While sensitivity did not differ significantly between subsites, specificity showed marked variation, resulting in substantial differences in overall diagnostic accuracy. Thus, subsite-related variation in CT performance appears to be driven primarily by differences in specificity rather than sensitivity. In particular, CT demonstrated very high specificity in tongue carcinomas (99.3%), indicating that false-positive findings are rare in this subsite. In contrast, specificity was substantially lower in tumors of the alveolar ridge, especially in the maxillary alveolar ridge (40.0%), suggesting a higher rate of false-positive interpretation in anatomically bone-adjacent regions. These findings can be explained by anatomical factors. Tumors arising in close proximity to cortical bone are more likely to induce reactive or structural bone changes that may mimic tumor infiltration on CT (Jimi et al. 2011; Handschel et al. 2012). Conversely, tumors of the tongue are typically separated from bone, and radiological signs of bone involvement therefore represent more advanced disease stages.

From a clinical perspective, these results support a more localization-aware interpretation of CT findings. In subsites with high specificity, such as the tongue, a positive CT finding may be considered highly reliable. In contrast, in subsites with lower specificity, particularly the alveolar ridge, radiological findings should be interpreted with caution to avoid potential overestimation of bone involvement (Czerwonka et al. 2017; DeAngelis et al. 2019).

Importantly, the absence of significant differences in sensitivity suggests that CT has a consistent limitation in detecting early or subtle bone invasion across subsites.

Taken together, these results support a localization-specific interpretation of CT findings rather than a uniform diagnostic approach. Exclusion of artifact-affected cases resulted in a modest improvement in diagnostic performance, underscoring the impact of image quality, even though anatomical factors might be the main drivers of diagnostic variability (Slieker et al. 2020; Struckmeier et al. 2024). Beyond the scope of the present study, technical developments such as artifact-reduction strategies and advances in CT acquisition may further improve diagnostic reliability (Mukaigawa et al. 2025; Kim et al. 2026). In addition, the complementary use of other imaging modalities, particularly magnetic resonance imaging (MRI), may be beneficial in selected cases with suspected bone involvement (Ye et al. 2024). However, these approaches require further prospective evaluation.

This study has several limitations. First, its retrospective single-center design may limit generalizability. Secondly, some anatomical subsites were represented by small sample sizes, so localization-specific estimates should be interpreted with caution. Finally, subtle radiological features such as periosteal thickening could not be systematically evaluated, as they were not consistently documented in the original reports.

## Conclusion

In conclusion, preoperative CT provides clinically relevant information for assessment of bone invasion in OSCC, but its diagnostic performance is highly dependent on tumor localization and radiological severity. While CT reliably excludes bone invasion in many cases, its diagnostic performance varies across anatomical subsites, mainly due to differences in specificity. A localization-aware interpretation of CT findings, combined with explicit reporting of radiological severity, may support preoperative decision-making.

## Supplementary Information

Below is the link to the electronic supplementary material.

Supplementary file1 (DOCX 27 kb)

## Data Availability

The datasets generated during and analysed during the current study are available from the corresponding author on reasonable request.

## Abbreviations

ASA: American society of anesthesiologists
AUC: Area under the curve
CI: Confidence interval
CT: Computed tomography
DOI: Depth of invasion
MRI: Magnetic resonance imaging
NPV: Negative predictive value
OSCC: Oral squamous cell carcinoma
PACS: Picture archiving and communication system
PPV: Positive predictive value
ROC: Receiver operating characteristic

## References

[CR1] Amin MB, Greene FL, Edge SB, Compton CC, Gershenwald JE, Brookland RK, Meyer L, Gress DM, Byrd DR, Winchester DP (2017) The eighth edition AJCC cancer staging manual: continuing to build a bridge from a population-based to a more “personalized” approach to cancer staging. CA Cancer J Clin 67:93–99 28094848 10.3322/caac.21388

[CR2] Arya S, Rane P, Deshmukh A (2014) Oral cavity squamous cell carcinoma: role of pretreatment imaging and its influence on management. Clin Radiol 69:916–930 24908285 10.1016/j.crad.2014.04.013

[CR3] Czerwonka L, Bissada E, Goldstein DP, Wood RE, Lam EW, Yu E, Lazinski D, Irish JC (2017) High-resolution cone-beam computed tomography for assessment of bone invasion in oral cancer: comparison with conventional computed tomography. Head Neck 39:2016–2020 28703386 10.1002/hed.24858

[CR4] DeAngelis A, Breik O, Angel C, Goh C, Iseli T, Nastri A, McCullough M, Wiesenfeld D (2019) Can radiological examination of mandibular bone invasion accurately predict the need for mandibular resection in oral squamous cell carcinoma? Int J Oral Maxillofac Surg 48:576–583 30594479 10.1016/j.ijom.2018.12.007

[CR5] Ebrahimi A, Murali R, Gao K, Elliott MS, Clark JR (2011) The prognostic and staging implications of bone invasion in oral squamous cell carcinoma. Cancer 117:4460–4467 21437887 10.1002/cncr.26032

[CR6] Fives C, Nae A, Roche P, O’Leary G, Fitzgerald B, Feeley L, Sheahan P (2017) Impact of mandibular invasion on prognosis in oral squamous cell carcinoma four centimeters or less in size. Laryngoscope 127:849–854 27481484 10.1002/lary.26211

[CR7] Gill N, Meena V, Shivkumar, (2025) Diagnostic accuracy of CT and MRI for mandibular invasion in oral cavity carcinoma: a histopathology-correlated study using Brown’s and Mcgregor’s criteria. Eur Arch Otorhinolaryngol 283(1):425–432 41243014 10.1007/s00405-025-09814-x

[CR8] Goerres GW, Schmid DT, Schuknecht B, Eyrich GK (2005) Bone invasion in patients with oral cavity cancer: comparison of conventional CT with PET/CT and SPECT/CT. Radiology 237:281–287 16118155 10.1148/radiol.2371041228

[CR9] Hakim SG, Wieker H, Trenkle T, Sieg P, Konitzer J, Holl-Ulrich K, Jacobsen HC (2014) Imaging of mandible invasion by oral squamous cell carcinoma using computed tomography, cone-beam computed tomography and bone scintigraphy with SPECT. Clin Oral Investig 18:961–967 23873323 10.1007/s00784-013-1042-z

[CR10] Handschel J, Naujoks C, Depprich RA, Kubler NR, Kropil P, Kuhlemann J, Jansen TM, Boeck I, Sproll KC (2012) CT-scan is a valuable tool to detect mandibular involvement in oral cancer patients. Oral Oncol 48:361–366 22155255 10.1016/j.oraloncology.2011.11.009

[CR11] Haseli S, Park C, Azhideh A, Karande G, Chalian M (2025) Performance and reliability comparison: original vs. revised bone reporting and data system (Bone-RADS). Skeletal Radiol 54:1681–1688 39838067 10.1007/s00256-025-04865-x

[CR12] Jimi E, Furuta H, Matsuo K, Tominaga K, Takahashi T, Nakanishi O (2011) The cellular and molecular mechanisms of bone invasion by oral squamous cell carcinoma. Oral Dis 17:462–468 21496184 10.1111/j.1601-0825.2010.01781.x

[CR13] Kim S, Kim SH, Song MK, Shin J, Seo JH, Kang JY (2026) New beam hardened data correction and its application to artifact reduction in CT images. Med Phys 53:e70292 41579111 10.1002/mp.70292 PMC12831530

[CR14] Kouketsu A, Miyashita H, Kojima I, Sakamoto M, Murata T, Mori S, Nogami S, Yamauchi K, Nagai H, Kumamoto H, Takahashi T (2021) Comparison of different diagnostic imaging techniques for the detection of bone invasion in oral cancers. Oral Oncol 120:105453 34265573 10.1016/j.oraloncology.2021.105453

[CR15] Lee C, Choi YJ, Jeon KJ, Kim DW, Nam W, Kim HJ, Cha IH, Han SS (2020) Prognostic implications of combined imaging and histologic criteria in squamous cell carcinoma with mandibular invasion. J Clin Med 9:2020 32375278 10.3390/jcm9051335 PMC7291115

[CR16] Li C, Lin J, Men Y, Yang W, Mi F, Li L (2017) Does medullary versus cortical invasion of the mandible affect prognosis in patients with oral squamous cell carcinoma? J Oral Maxillofac Surg 75:403–415 27621147 10.1016/j.joms.2016.08.005

[CR17] Li C, Men Y, Yang W, Pan J, Sun J, Li L (2014) Computed tomography for the diagnosis of mandibular invasion caused by head and neck cancer: a systematic review comparing contrast-enhanced and plain computed tomography. J Oral Maxillofac Surg 72:1601–1615 24679956 10.1016/j.joms.2014.02.014

[CR18] Mukaigawa T, Asakura K, Tsuzuki A, Urikura A, Yoshida T, Goto S, Okada S, Hiiragi Y, Sato F (2025) Subtraction CT improves detectability of mandibular bone invasion in oral squamous cell carcinoma. Laryngoscope 135:1706–1714 39651678 10.1002/lary.31946

[CR19] Okura M, Yanamoto S, Umeda M, Otsuru M, Ota Y, Kurita H, Kamata T, Kirita T, Yamakawa N, Yamashita T, Ueda M, Komori T, Hasegawa T, Aikawa T (2016) Japan oral oncology G: prognostic and staging implications of mandibular canal invasion in lower gingival squamous cell carcinoma. Cancer Med 5:3378–3385 27758080 10.1002/cam4.899 PMC5224841

[CR20] Onoue K, Yakami M, Nishio M, Sakamoto R, Aoyama G, Nakagomi K, Iizuka Y, Kubo T, Emoto Y, Akasaka T, Satoh K, Yamamoto H, Isoda H, Togashi K (2021) Temporal subtraction CT with nonrigid image registration improves detection of bone metastases by radiologists: results of a large-scale observer study. Sci Rep 11:18422 34531429 10.1038/s41598-021-97607-7 PMC8446090

[CR21] Ritschl LM, Niu M, Sackerer V, Classen C, Stimmer H, Fichter AM, Wolff KD, Grill FD (2023) Effect of segmental versus marginal mandibular resection on local and lymph node recurrences in oral squamous cell carcinoma: is tumorous bone infiltration or location and resulting soft tissue recurrences a long-term problem? J Cancer Res Clin Oncol 149:11093–11103 37344607 10.1007/s00432-023-04963-0 PMC10465630

[CR22] Slieker FJB, Dankbaar JW, de Bree R, Van Cann EM (2020) Detecting bone invasion of the maxilla by oral squamous cell carcinoma: diagnostic accuracy of preoperative computed tomography versus magnetic resonance imaging. J Oral Maxillofac Surg 78:1645–1652 32445627 10.1016/j.joms.2020.04.019

[CR23] Struckmeier AK, Buchbender M, Agaimy A, Kesting M (2024) Diagnostic accuracy of contrast-enhanced computed tomography in assessing bone invasion in patients with oral squamous cell carcinoma. Clin Oral Investig 28:314 38748270 10.1007/s00784-024-05705-3 PMC11096202

[CR24] Timmer V, Crombag G, van Kuijk SMJ, Vaassen LAA, Kessler P, Postma AA (2025) The accuracy of dual energy CT on evaluation of bone invasion caused by oral squamous cell carcinoma - a comparison to MRI. J Craniomaxillofac Surg 53:1731–1737 40753038 10.1016/j.jcms.2025.07.005

[CR25] Tudor GR, Finlay DB (1999) Is there an improvement in performance when radiographs are re-reported at 24 hours? Br J Radiol 72:465–468 10505011 10.1259/bjr.72.857.10505011

[CR26] Uribe S, Rojas LA, Rosas CF (2013) Accuracy of imaging methods for detection of bone tissue invasion in patients with oral squamous cell carcinoma. Dentomaxillofac Radiol 42:20120346 23420854 10.1259/dmfr.20120346 PMC3667522

[CR27] Ye Y, Zheng X, Chen T, Zheng K, Pan J, Lin L (2024) Computed tomography/magnetic resonance imaging for mandibular boundary invasion of oral squamous cell carcinoma assessment. BMC Oral Health 24:172 38308269 10.1186/s12903-024-03920-8 PMC10837888

