# Supplementary material for: Preoperative computed tomography for assessment of bone invasion in oral squamous cell carcinoma: diagnostic accuracy and anatomical subsite dependency
Source: J Cancer Res Clin Oncol. 2026 Apr 22;152(4):91. doi: 10.1007/s00432-026-06472-2 (PMC13103028; doi:10.1007/s00432-026-06472-2)
Supplement: Supplementary file 1 — Supplementary file1 (DOCX 27 kb) [file 432_2026_6472_MOESM1_ESM.docx]

# Preoperative Computed Tomography for Assessment of Bone Invasion in Oral Squamous Cell Carcinoma: Diagnostic Accuracy and Anatomical Subsite Dependency

Jannik Ketschau MD ^1^; Yannik Leonhardt MD ^2^; Alex Grabenhorst MD DMD ^1^; Hannes Singer MD DMD ^1^; Helena Kram MD ^1^; Nils Krautkremer MD DMD ^1^; Katharina Pippich MD DMD ^1^; Herbert Stimmer ^2^; Klaus-Dietrich Wolff MD DMD ^1^; Jonathan Mohr MD DMD ^1^; Lucas M. Ritschl MD DMD ^1^

^1^ Department of Oral and Maxillofacial Surgery, TUM University Hospital Klinikum Rechts der Isar, School of Medicine and Health, Technical University of Munich, Germany

^2^ Institut of Diagnostic und Interventional Radiology, TUM University Hospital Klinikum rechts der Isar, School of Medicine and Health, Technical University of Munich, Germany

**Appendix**

**Corresponding author:**

Jannik Ketschau

Department of Oral and Maxillofacial Surgery

TUM University Hospital Klinikum Rechts der Isar

School of Medicine and Health

Technical University of Munich

Ismaninger Strasse 22, D-81675 Munich, Germany

Email: [jannik.ketschau@tum.de](mailto:jannik.ketschau@tum.de)

# Supplementary table S1: Distribution of histologically confirmed bone invasion by tumor localization

| **Tumor localization** | **Bone invasion yes** | **Bone invasion no** | **Total** |
| --- | --- | --- | --- |
| Floor of mouth | 49 (23.1%) | 163 (76.9%) | 212 |
| Tongue | 4 (2.6%) | 151 (97.4%) | 155 |
| Mandibular alveolar ridge | 50 (51.0%) | 48 (49.0%) | 98 |
| Buccal mucosa | 7 (15.3%) | 39 (84.7%) | 46 |
| Retromolar region | 5 (27.8%) | 13 (72.2%) | 18 |
| Maxillary alveolar ridge | 17 (77.3%) | 5 (22.7%) | 22 |
| Hard palate | 1 (16.7%) | 5 (83.3%) | 6 |
| Soft palate | 1 (6.7%) | 14 (93.3%) | 15 |

Table S1: Distribution of histologically confirmed bone invasion across anatomical subsites of oral squamous cell carcinoma. Values are presented as number and percentage within each tumor localization. Histopathology served as the reference standard for assessment of bone invasion.

# Supplementary table S2: Radiological CT findings by tumor localization

| **Tumor localization** | **CT negative** | **CT positive** | **Total** |
| --- | --- | --- | --- |
| Floor of mouth | 168 (79.2%) | 44 (20.8%) | 212 |
| Tongue | 153 (98.7%) | 2 (1.3%) | 155 |
| Mandibular alveolar ridge | 49 (50.0%) | 49 (50.0%) | 98 |
| Buccal mucosa | 38 (82.6%) | 8 (17.4%) | 46 |
| Retromolar region | 15 (83.3%) | 3 (16.7%) | 18 |
| Maxillary alveolar ridge | 7 (31.8%) | 15 (68.2%) | 22 |
| Hard palate | 5 (83.3%) | 1 (16.7%) | 6 |
| Soft palate | 12 (80.0%) | 3 (20.0%) | 15 |

Table S2: Distribution of dichotomized radiological CT assessment of bone involvement (negative vs. positive) across anatomical subsites. Values are presented as number and percentage within each tumor localization.

# Supplementary table S3: Radiological severity and probability of histologically confirmed bone invasion

| **Radiological grading** | **Bone invasion yes** | **Bone invasion no** | **Total** |
| --- | --- | --- | --- |
| No bone invasion / not mentioned | 49 (11.1%) | 394 (88.9%) | 443 |
| Cortical erosion | 25 (49.0%) | 26 (51.0%) | 51 |
| Bone destruction | 60 (81.1%) | 14 (18.9%) | 74 |

Table S3: Relationship between radiological CT grading of bone involvement and histologically confirmed bone invasion. Radiological severity was categorized as no bone invasion or not mentioned, cortical erosion, and overt bone destruction. Percentages indicate the proportion of cases with histologically confirmed bone invasion within each radiological severity category.

# Supplementary table S4: Diagnostic performance after exclusion of imaging artifacts

| **Diagnostic parameter** | **Value** |
| --- | --- |
| Number of cases | 453 |
| Sensitivity | 68.9% |
| Specificity | 87.9% |
| Positive predictive value (PPV) | 67.7% |
| Negative predictive value (NPV) | 88.4% |
| AUC (ROC analysis) | 0.80 |

Table S4: Diagnostic accuracy of preoperative CT for detection of bone invasion after exclusion of cases with relevant imaging artifacts. Sensitivity, specificity, positive predictive value (PPV), negative predictive value (NPV), and area under the receiver operating characteristic curve (AUC) are reported.
